# Interfacing MXene Flakes on a Magnetic Fiber Network as a Stretchable, Flexible, Electromagnetic Shielding Fabric

**DOI:** 10.3390/nano12010020

**Published:** 2021-12-22

**Authors:** Zhen Miao, Xiaohong Chen, Honglei Zhou, Ping Liu, Shaoli Fu, Jiajie Yang, Yuhang Gao, Yupeng Ren, Dong Rong

**Affiliations:** School of Materials Science and Engineering, University of Shanghai for Science and Technology, Shanghai 200000, China; 193742704@st.usst.edu.cn (Z.M.); 193742699@st.usst.edu.cn (H.Z.); 193742700@st.usst.edu.cn (P.L.); 171380130@st.usst.edu.cn (S.F.); 192432667@st.usst.edu.cn (J.Y.); 193742737@st.usst.edu.cn (Y.G.); 192432629@st.usst.edu.cn (Y.R.); 193742742@st.usst.edu.cn (D.R.)

**Keywords:** electrospun film, structural design, electromagnetic synergy, flexible and reliable, shielding mechanism

## Abstract

A unique self-standing membrane composed of hierarchical thermoplastic polyurethane (TPU)/polyacrylonitrile (PAN) fibers is prepared by the electrospinning technique, followed by a simple dip-coating process. Fe_3_O_4_ nanoparticles are uniformly anchored on TPU/PAN fibers during the electrospinning process, enabling the membrane to achieve effective electromagnetic interference shielding (EMI SE) performance. Such a hybrid membrane has a high magnetization of 18.9 emu/g. When MXene (Ti_3_C_2_T*_x_*) layers are further loaded on the TPU/PAN/Fe_3_O_4_NPs hybrid membrane, its EMI SE performance in the X band can exceed 30 dB due to the hydrogen bonds generated between the macromolecular chain of PAN and the functional group (T*_x_*) on the surface of MXene. Simultaneously, the interfacial attraction between MXene and the TPU/PAN/Fe_3_O_4_NPs substrate is enhanced. The EMI SE mechanism of the hybrid membrane indicates that this film has great potential in the fields of wearable devices and flexible materials

## 1. Introduction

The continuous development of the intelligence level of wireless communications technology prompts a heavy use of flexible electronic equipment. Enormous quantities of such electronic equipment will produce a large amount of electromagnetic pollution, which can disturb high-precision electronic devices, and poses potential human health hazards. To address this problem, the development of high-performance shielding materials is urgently required in order to prevent or minimize harmful radiation [1,2,3,4,5]. Currently, many studies in this field are focusing on the preparation of electromagnetic interference (EMI) shielding fabrics via the integration of metal particles and polymeric or inorganic fabrics [6,7,8]. Such EMI shielding fabrics have excellent conductivity and effective protection performance. Nevertheless, the cost to fabricate such EMI shielding fabrics is very high, and their life span is short due to their susceptibility to migration under humid and high-temperature environments. In contrast to traditional metal materials, Fe_3_O_4_ nanoparticles have recently been introduced into EMI shielding fabrics as mixed fillers to enhance electromagnetic wave absorption by making use of their high magnetic properties and excellent compatibility with polymeric materials. However, it is still a huge challenge to overcome the agglomeration and uncontrollable loading of Fe_3_O_4_ nanoparticles on the fabrics [9,10]. Carbon-based EMI shielding materials usually have a large surface-to-volume ratio, high chemical and thermal stability, good electrical conductivity, and high EMI *SE*; such materials include reduced graphene [11], carbon foam [12], multiwalled carbon nanotubes [13], carbon nanofibers [14], etc. Nevertheless, to the best of our knowledge, no EMI *SE* materials have been reported with a combination of extremely small thickness, high flexibility, and excellent EMI *SE* properties. MXenes are two-dimensional metal carbide/nitride layered nanomaterials. Due to their unique layered structure and metal-like conductivity, MXenes have been extensively researched in the field of electromagnetic shielding [15,16,17,18,19]. MXene materials are obtained by selectively etching away the Al layer from a MAX phase, and they have the structure M*_n_*_+1_X*_n_*T*_x_*. Specifically, M is a transition metal element, X is carbon and/or nitrogen, and T*_x_* is the terminating group (–F, =O, or –OH) [20,21]. Compared with other two-dimensional materials that lack hydrophilicity, MXenes can be used to easily obtain composite films or fabrics through self-assembly, vacuum filtration, dip coating, and other methods [22,23,24,25,26,27]. However, the relatively weak interaction between the hydrophilic surface of MXenes and hydrophobic elastic matrices limits their application in wearable devices.

Moreover, the reflection of EM waves directly generated on the surface of these materials will inevitably cause secondary electromagnetic radiation pollution and affect the stability and accuracy of devices [28,29]. The prevalence of electromagnetic shielding fabrics has sharply increased due to their light weight, high efficiency, flexibility, customizable molding, and application in complex scenarios [30,31,32,33]. Therefore, new strategies to construct complex conductive networks with adjustable electromagnetic properties should be studied in order to achieve a shielding mechanism based on absorption. MXene coatings can strongly and reliably interact with substrates, showing promise for significantly expanding their use in electromagnetic shielding fabric applications.

This article demonstrates the construction of a conductive and magnetic dual-functional gradient electromagnetic shielding network in the structure of electrospun TPU/PAN/Fe_3_O_4_NPs fibers by soaking and drying in a simple solution. Thermoplastic polyurethane (TPU) matrix materials have gained great attention due to the advantages of easy processing and beneficial mechanical properties. Here, we chose TPU as the matrix material; TPU/PAN/Fe_3_O_4_NPs composite film was prepared by electrospinning, with Fe_3_O_4_NPs as a functional filler and PAN as a binder to increase the interaction between the matrix material and the MXene coating. The reflection characteristics of TPFM composite film after dip coating with MXene are affected by the asymmetric network, and the average reflection efficiency (*SE_R_*) is as low as 4.8 dB. Unlike modifying the surface of MXene with electrically insulating organic molecules to improve the interaction between the MXene and the substrate, the introduction of PAN and Fe_3_O_4_NPs via co-spinning maintains the excellent conductivity of the MXene, keeping the MXene nanosheets firmly “locked” on the surface of the modified fiber to achieve high dispersion. In contrast to the use of simple stacking strategies, the MXene nanosheets were very tightly bound to the fibers. Moreover, the prepared TPU/PAN/Fe_3_O_4_/MXene film exhibited stable mechanical properties. The exceptional properties of the obtained composite fabrics make them suitable for EMI shielding in wireless technology, robotic joints, and flexible wearable electronic devices.

## 2. Materials and Methods

### 2.1. Materials

Thermoplastic polyurethane (TPU) granules (Elastollan960A) were supplied by BASF Co., Ltd., Ludwigshafen, Germany. Ti_3_AlC_2_ was purchased from 11 Technology Co., Ltd., Jilin, China. PAN powder (Mw = 90,000) was purchased from Sinopharm Chemical Reagent Co., Ltd., Shanghai, China. Analytically pure hydrochloric acid (HCl) and lithium fluoride (LiF) were provided by Sinopharm Chemical Reagent Co., Ltd. N, *N*-dimethylformamide (DMF) and acetone were purchased from Sinopharm Chemical Reagent Co., Ltd. Fe_3_O_4_NPs were purchased from Sigma Co., Ltd., St. Louis, MO, USA. All materials were used as received, without any further purification.

### 2.2. MXene Solution Preparation

Ti_3_C_2_T*_x_* MXene was prepared by etching aluminum from Ti_3_AlC_2_ powder with a modified clay method. In brief, 2 g of LiF and 40 mL of HCl (9M) were stirred in a Teflon vessel for 30 min. Next, 2 g of Ti_3_AlC_2_ was slowly added to the beaker under continuous stirring at 35 °C for 24 h. The mixture was then centrifuged until the pH value was greater than 6. The final precipitate was dispersed in deionized water and sonicated in an ice bath for 1 h. Then, it was centrifuged at 3500 rpm for 1 h, and the black/brown-colored supernatant was collected as a small-layer dispersion liquid. The uniformly dispersed supernatant exhibited the Tyndall effect (Figure S1c).

### 2.3. Preparation of TPU/PAN/Fe_3_O_4_/MXene Films

**Figure 1:**
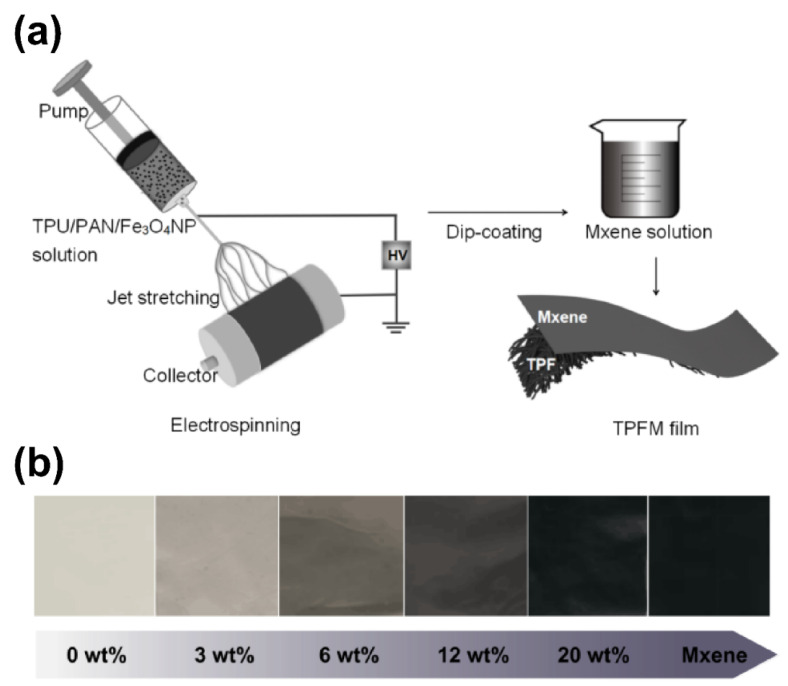
(**a**) Schematic diagram of the fabrication of the TPU/PAN/Fe_3_O_4_/MXene fabric. (**b**) Optical images of electrospun mats with increasing concentration of Fe_3_O_4_NPs from 0 to 10 wt%.

The manufacturing process for obtaining the TPU/PAN/Fe_3_O_4_/MXene (TPFM) films is shown in Figure 1a. Colloidal solutions were prepared by dissolving 14 wt% TPU/PAN (TPU:PAN = 5:2) in a mixed solution containing DMF and acetone at a mass ratio of 3:1. The increased volatility of this solvent was necessary in order to reduce the formation of a large number of beads during the electrospinning process. Next, different weight ratios of Fe_3_O_4_NPs (0, 3, 6, 12, and 20 wt%) were dispersed in this solution. The electrospinning solution was then mechanically stirred at room temperature for 12 h.

The prepared electrospinning solution was loaded into a plastic syringe with an 18G metal needle. A positive voltage (15 kV) was applied to the needle tip, and the collector plate was grounded by covering it with aluminum foil. The distance between the needle tip and the collector was 15 cm, and the infusion rate was 1–1.5 mL/h. All samples were electrospun under the conditions of 30–35% relative humidity and 25 ± 2 °C. The fiber membranes were then separated from the aluminum foil and dried for 12 h. These fiber membranes were denoted TPF_0_, TPF_3_, TPF_6_, TPF_12_, and TPF_20_ based on their Fe_3_O_4_NP contents. Next, the TPU/PAN/Fe_3_O_4_ (TPF) films with different Fe_3_O_4_NP contents were soaked in a deionized water solution containing MXene (2 mg/mL or 5 mg/mL). After drying at 50 °C for 1 h, the MXene sheets formed a uniform coating structure on the TPF films; this structure was the TPFM mat. Figure 1b shows digital photographs of TPF films with different nanomagnetic particle contents and TPF films coated with MXene.

### 2.4. Characterization

The morphology of the nanomaterials was observed via FE-SEM (FEI, Quanta FEG 450, Hillsboro, OR, USA), and their crystal structure was characterized by XRD (Bruker, D8 Advance, Berlin, Germany); their chemical states were identified by X-ray photoelectron spectroscopy (XPS, Thermo Scientific, ESCALAB 250, Waltham, MA, USA). FTIR spectra were taken for a uniformly dispersed sample in KBr in the compressed pellet form, using a PerkinElmer RXI FTIR spectrometer (Waltham, MA, USA) in the frequency range of 500–4000 cm^−1^.

The electrical conductivity values of the TPU/PAN/Fe_3_O_4_/MXene films obtained under different preparation conditions were tested using the four-point probe method at room temperature. Each reported measurement is the average of four samples. The details are reported in our previous work.

A vibrating sample magnetometer (VSM, MicroSense, EV9, Lowell, MA, USA) was used to measure magnetic properties at 300 K. The EMI shielding performance of the sample was measured with an Agilent N5232A vector network analyzer in the 8–12 GHz (X band) microwave range, using waveguide calibration (HD-100VNAWKS, Santa Clara, CA, USA). The measured scattering parameters (*S*_11_ and *S*_12_) were used to calculate the EMI *SE*. The specific calculation formulae for determining total EMI *SE* (*SE_T_*), absorption shielding effectiveness (*SE_A_*), reflection (*SE_R_*), and multiple internal reflection (*SE_M_*) are as follows [34,35]:

$$R={\left|S_{11}\right|}^{2}$$

$$T={\left|S_{12}\right|}^{2}$$

R+A+T=1

$$SE_{R}=10log\left(\frac{1}{1-R}\right)=10log\left(\frac{1}{1-{\left|S_{11}\right|}^{2}}\right)$$

$$SE_{A}=10log\left(\frac{1}{1-A}\right)=10log\left(\frac{1-{\left|S_{11}\right|}^{2}}{{\left|S_{12}\right|}^{2}}\right)$$

$$SE_{T}=SE_{R}+SE_{A}+SE_{M}$$

where *T*, *R*, and *A* represent the transmission coefficient, reflection, and absorption coefficient, respectively. When *SE_T_* ≥ 15 dB, the *SE_M_* can be negligible [11,28].

## 3. Results and Discussion

**Figure 2:**
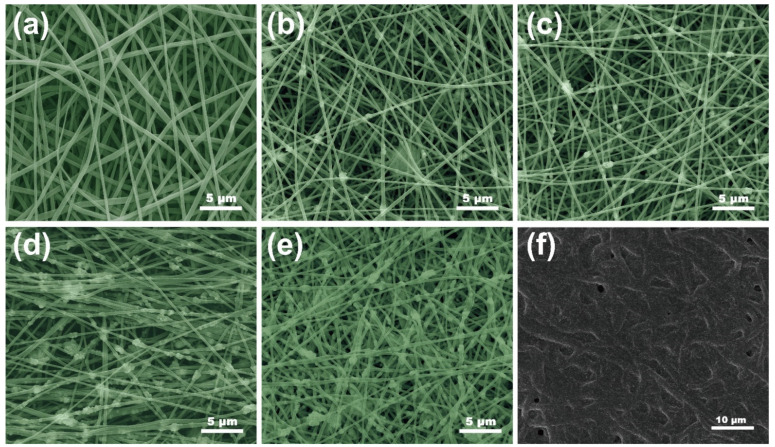
FE-SEM images of the electrospun composite films with (**a**) 0, (**b**) 3, (**c**) 6, (**d**) 12, and (**e**) 20 wt % Fe_3_O_4_NP contents. (**f**) TPFM film.

Figure 2 shows FE-SEM images displaying the microscopic morphology of the fabricated nanocomposite fabrics with different Fe_3_O_4_NP contents. These nanocomposite fiber membranes consist of a three-dimensional network structure formed by interspersed superfine fibers, and the magnetic nanoparticles are randomly distributed throughout the fibers. As shown in Figure 2b–e, due to the stable formation of Taylor cones and the high volatility of the precursor liquid, the TPF nanofiber membranes have no defects (such as spindles or beads). The viscosity of the precursor fluid decreases as the magnetic nanoparticle content increases; this helps the electrostatic force on the surface of the Taylor cone to overcome the viscous resistance, making the electrospinning jet thinner, and reducing the diameter of the produced fibers. In addition, as the Fe_3_O_4_NP content increases, Fe_3_O_4_NPs partially agglomerate in the solution, resulting in varying degrees of bulging on the fibers. Under this condition, the jet is not stable under the action of the electric field, resulting in an uneven fiber surface; this is conducive to microwave energy loss. As shown in Figure 2f, the MXene sheet is continuously and uniformly dispersed on the surface of TPU/PAN/Fe_3_O_4_ with a “scale” appearance, demonstrating that the fibers have thickened and filled into a fiber network. At the same time, the nanofiber network structure can be observed. The element mapping images of TPFM displayed in Figure S2 also show that the MXene layer is evenly dispersed on the fiber surface.

**Figure 3:**
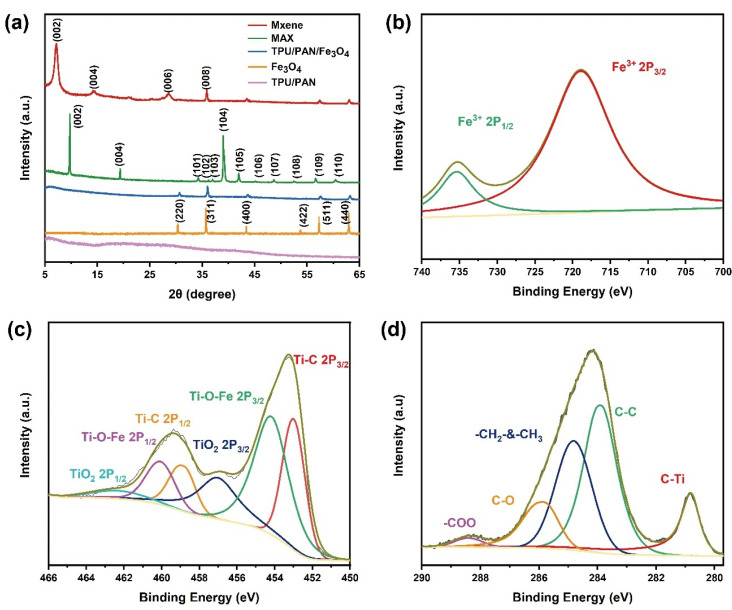
(**a**) Normalized XRD patterns of pure Fe_3_O_4_NPs, TPU/PAN film, and TPF film. (**b**–**d**) High-resolution XPS spectra of TPFM in the (**b**) Fe 2p, (**c**) Ti 2p, and (**d**) C 1s regions.

The X-ray diffraction (XRD) patterns of the specimens are shown in Figure 3. As shown in Figure 3a, the diffraction peak of MXene obtained after selective etching is at 2θ = 39°, and the most substantial diffraction peak of the MAX phase disappears; this indicates that the etching was successful. The peaks at 2θ = 8.90°, 18.24°, and 27.65° are attributed to the (002), (006), and (008) crystal planes, respectively. In addition, the (002) peak shifts to a lower angle due to the lattice expansion caused by the attachment of groups such as –F or –OH groups to the surface of the MXene. Moreover, this peak is lower in intensity, indicating electrospinning; this indicates the lower re-agglomeration of the Ti_3_C_2_ nanosheets. The XRD pattern of the Fe_3_O_4_NPs displays characteristic face-centered cubic Fe_3_O_4_ peaks at 30.2°, 35.4°, 43.7°, 56.9°, and 63.1°, which are attributed to its (220), (311), (400), (511), and (440) planes, respectively. Because the phase structure of the composite nanofiber textile material does not change, the XRD diffraction patterns of all of the TPFM composite nanofibers with different Fe_3_O_4_NP contents show similar XRD diffraction patterns. Due to the encapsulation of TPU/PAN, the relative intensities of their diffraction peaks are reduced compared with the diffraction pattern of pure Fe_3_O_4_NPs [36]. In addition, no characteristic diffraction peaks of any other impurities are visible.

To determine the elemental composition of TPFM and investigate the nature of its surface functional groups, X-ray photoelectron spectroscopy (XPS) was performed, and the results were analyzed with Gaussian–Lorentzian curve peak fitting calculations. The Ti 2p XPS spectrum of TPFM is shown in Figure 3c. The area ratio of the three fixed dual-level peaks (Ti 2p_1/2_, Ti 2p_3/2_) at 452.9, 454.3, and 457.1 eV is 2:1; these peaks correspond to Ti–C, Ti–O–Fe, and TiO_2_, respectively, and they demonstrate the strong interaction between them [37]. The Fe 2p XPS spectrum of TPFM is shown in Figure 3b. The peaks at 718.73 eV and 735.58 eV correspond to Fe 2p_3/2_ and Fe 2p_1/2_, respectively [38]. The C1s XPS spectrum shown in Figure 3d proves the presence of C–Ti (280.8 eV), C–C (283.9 eV), –CH_2_– and –CH_3_ (284.83 eV), CO (285.93 eV), and –COO (288.43 eV) [39].

**Figure 4:**
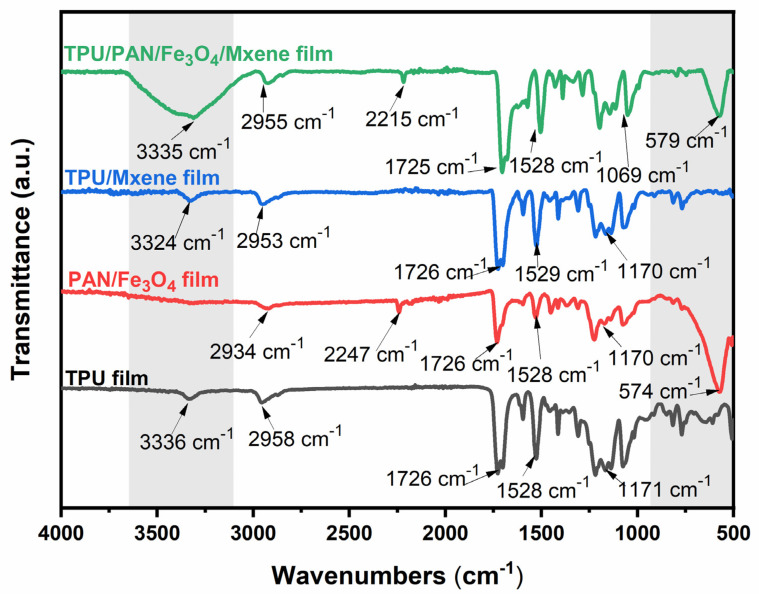
FTIR spectra of TPU, PAN/Fe_3_O_4_, TPU/MXene, and PAN/Fe_3_O_4_/MXene films.

The FTIR spectra of the samples are shown in Figure 4. The peaks at 3336 cm^−1^ and 2958 cm^−1^ in the FTIR spectrum of the original TPU fiber membrane are attributed to N–H stretching vibration and carbon–hydrogen polar covalent bond C–H stretching vibration, respectively [40]. The peak at 1726 cm^−1^ is the urethane bond. The peaks at 1528 cm^−1^ and 1171 cm^−1^ are caused by N–H in-plane bending vibration and C–O stretching vibration, respectively [41]. There is no significant shift in the TPU/MXene spectrum compared to the TPU spectral peak, reflecting that TPU and MXene only weakly interact. An analysis of the sudden disappearance of the PAN/Fe_3_O_4_ film signal in the 3431 cm^−1^ band clearly shows that the nanoparticles strongly interact with nitrogen, hydrogen, carbon, and oxygen atoms in the PAN chain [42]. Moreover, the PAN/Fe_3_O_4_ film peak at 2247 cm^−1^ is attributed to –CN stretching vibration. However, the –CN peak in the PAN/Fe_3_O_4_NPs/MXene film spectrum is shifted to 2209 cm^−1^, and also demonstrates a broader peak shape. The –OH stretching vibration peak of MXene shifts to 3335 cm^−1^, indicating the formation of a hydrogen bond between –CN and –OH [43]. The changes in the adhesive strength of PAN/Fe_3_O_4_NPs/MXene, indicated by the peaks at 1457 cm^−1^, 1359 cm^−1^, 828 cm^−1^, and 767 cm^−1^, also imply the existence of a force. Some peaks are observed at a low wavenumber of 574 cm^−1^, indicating the presence of Fe_3_O_4_NPs in the PAN/Fe_3_O_4_ and PAN/Fe_3_O_4_/MXene films [44]. Overall, the addition of PAN and nanomagnetic particles improves interfacial interaction and coating dispersion, due to the hydrogen bonding and electrostatic interaction between the MXene and nanofibers.

**Figure 5:**
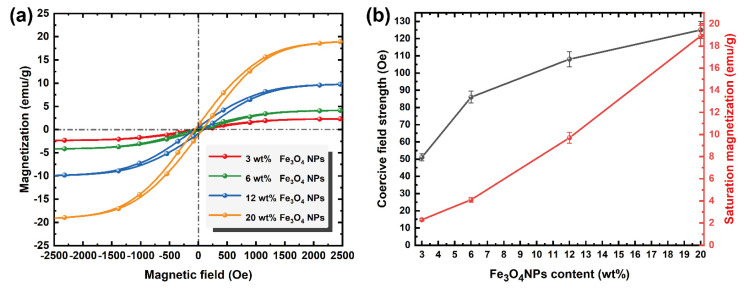
(**a**) Magnetization hysteresis loops of TPF. (**b**) Saturation magnetization and coercivity with the variation in the Fe_3_O_4_NP contents.

The magnetic properties of TPF were analyzed using a vibrating sample magnetometer at room temperature. TPF shows superparamagnetism due to the existence of the nanosized Fe_3_O_4_NPs. As shown in Figure 5b, with increasing Fe_3_O_4_NP content, the saturation magnetization increases linearly from 2.3 emu/g to 18.9 emu/g, and the coercive field strength increases linearly from 51 Oe to 125 Oe. As displayed in Figure 5a, the strong coercivity of TPF nanofilms at ~300 Oe is attributed to the shape anisotropy of the nanofiber and the ferromagnetic Fe_3_O_4_NPs acting as one domain. The magnetocrystalline anisotropy in the nanofiber films can generate more substantial magnetic cleaning and lead to higher coercivity [45].

**Figure 6:**
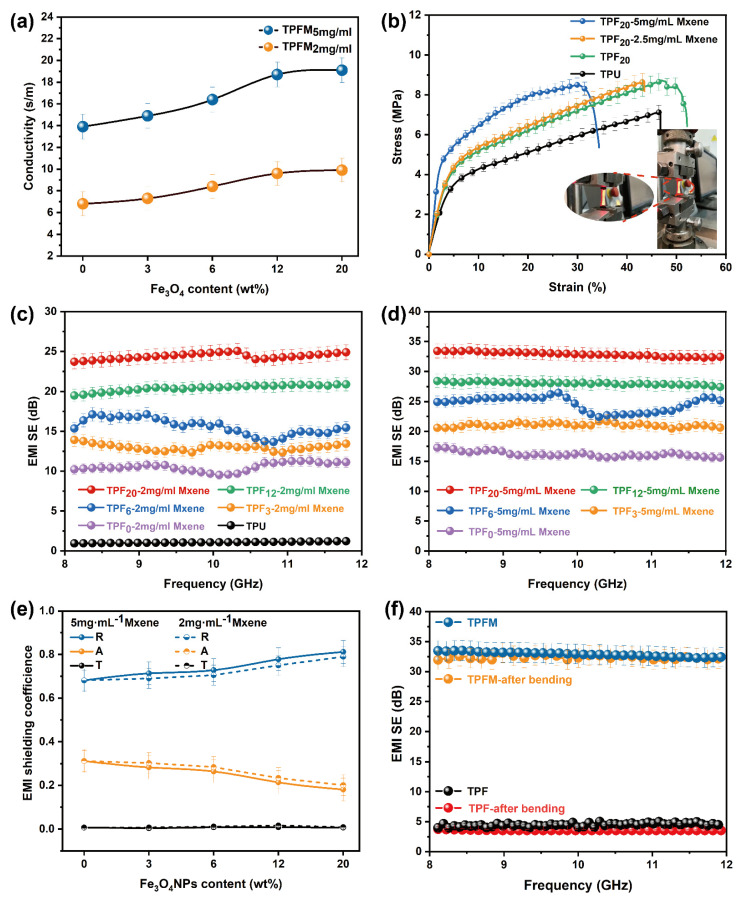
(**a**) The resistance of TPFM with different MXene concentrations in Fe_3_O_4_NPs. (**b**) The tensile properties of TPU, TPF, and TPFM. The inset shows a schematic of the microstrain–stress measurement system. (**c**,**d**) EMI *SE* of TPFM with different concentrations of Fe_3_O_4_NPs. (**e**) *R*, *A*, and *T* coefficients in the 8–12 GHz range. (**f**) EMI *SE* performance of TPFM after 500 fatigue cycle tests.

Relatively high electrical properties are an important condition for efficient EMI shielding performance. Therefore, electrical conductivity testing of composite nanofibers with different Fe_3_O_4_NP loadings was carried out with a 4-point probe device. As shown in Figure 6a, the electrical conductivity reached a maximum of 18.9 S/m. Changes in the electrical conductivity of these nanofibers can be attributed to the different Fe_3_O_4_ loadings. With increasing Fe_3_O_4_NP contents, the dispersion of MXene on the fiber surface is enhanced, the physical connections between the fibers are improved, and the MXene sheet layer forms a conductive path. Figure 6b shows the tensile properties of the composite fibers produced under different processes. The tensile stress of the pure TPU film is 5.78 MPa. When a quantitative PAN and 20 wt% Fe_3_O_4_NPs are added, the tensile stress increases to 8.65 MPa; the tensile curves of the composite films fabricated with different Fe_3_O_4_NP loadings are shown in Figure S3. As the magnetic nanoparticle loading increases, the tensile stress of TPF first increases and then decreases. Tensile strains lower than that of the initial TPU film are also obtained, which is due to the dispersion-strengthening effect of the Fe_3_O_4_NPs. This strengthening mechanism explains that a large number of Fe_3_O_4_NPs are attached to the TPU/PAN fiber membrane, which increases the roughness of the fiber membrane. Consequently, friction is generated between the fibers during stretching, increasing the stretching resistance. The applied load therefore increases the strength through the transfer of magnetic particles. The TPFM stretching curve clearly shows that MXene transmits some of the tensile force on the surface layer during stretching, which increases the tensile breaking strength but slightly reduces the tensile strain compared with the original fiber membrane. The effect of Fe_3_O_4_NPs on microwave attenuation has been repeatedly proven [46,47], Figure 6c,d show the EMI of the pure TPU film and the TPFM fiber films with different MXene concentrations and Fe_3_O_4_NP contents at 8–12 GHz (the X band). The pure TPU film has no shielding ability against electromagnetic waves, while TPU/PAN/Fe_3_O_4_/MXene shows satisfactory EMI shielding ability. With an Fe_3_O_4_NP loading of 20 wt%, the EMI *SE* values of films fabricated with 2 mg/mL and 5 mg/mL MXene increase to 24.9 dB (TPF_5_-2 mg/mL) and 32.5 dB (TPF_5_-5 mg/mL), respectively. To more reasonably evaluate the contribution of Fe_3_O_4_NPs to electromagnetic wave attenuation, the power coefficients *R*, *A*, and *T* were studied (Figure 6e). With increasing magnetic nanoparticle loading, *R* increases and *A* slightly decreases, proving that the addition of magnetic particles has a positive effect on EMI *SE*. To measure the stability of the material’s shielding performance, TPFM was tested for 500 cycles at room temperature. As shown in Figure 6f, the EMI *SE* of this TPFM was slightly reduced by 1.02% after 500 repeated bending tests; this was due to a small amount of Fe_3_O_4_NPs detaching from the fibers, as well as the destruction of the MXene conductive network.

**Figure 7:**
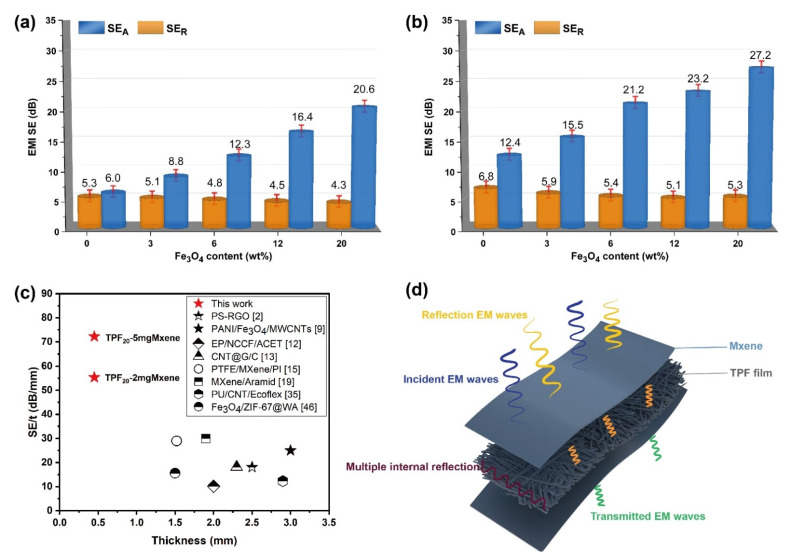
(**a**,**b**) Average EMI *SE SE_T_*, *SE_A_*, and *SE_R_* values of TPFM_2mg/mL_ and TPFM_5mg/mL_ at different Fe_3_O_4_NP contents. (**c**) Comparison of the *SE*/*t* of various fabric- and film-based shielding materials. (**d**) Schematic diagram of the electromagnetic shielding mechanism for TPFM.

Figure 7a,b show the average *SE_A_* and *SE_R_* values of films with Fe_3_O_4_NP loadings at different MXene concentrations. It can be seen that the value of *SE_A_* is much greater than SE_R_, which is due to the coordination effect of the entire shielding system. The adhesion of the MXene sheet significantly improves the EMI *SE* of the TPFM film. In Figure 7b, the surface reflection of TPF_20_-5 mg MXene is enhanced. This phenomenon can be attributed to the agglomeration of Fe_3_O_4_NPs due to the large Fe_3_O_4_NPs/MXene content. The internal electron spins of Fe_3_O_4_NPs can be arranged spontaneously in a small range to form a spontaneous magnetization zone. Besides, the double-layer non-magnetic coating on the surface of the Fe_3_O_4_ nanoparticles enhances the spin disorder in the TPFM composite material, and affects the transmission of microcurrents. Therefore, spin disturbance will affect the generation of microwave currents and affect the surface reflection. Figure 7c shows the *SE*/*t* comparison between the TPFM film and some EMI shielding materials reported in a previous work, where *SE*/*t* represents the shielding effect of the conductive material to remove the thickness factor. According to observations, the *SE*/*t* of the TPFM film can be as high as 72.5 (dB/mm), which achieves a high shielding effect at a very thin thickness.

Figure 7d (mechanism diagram) shows the coordination effect of the entire shielding system. In the absence of Fe_3_O_4_NPs, the absorption and attenuation of MXene to electromagnetic waves is relatively weak, because spin disorder plays a vital role in the absorption phenomenon of EMI shielding applications. Similarly, without the MXene interface, it is difficult to transmit the microcurrent generated by Fe_3_O_4_NPs. The surface functional groups and local defects of MXene after etching will produce dipolar polarization, which plays a key role in the dielectric loss [48]. At the same time, MXene’s multilayer structure provides a large number of interfaces, which is conducive to interface polarization. Secondly, the bridging effect of TPFM with high conductivity can generate more conductive paths for the movement of charges, which is conducive to the loss of conductivity [48]. When electromagnetic waves fall on the TPFM material, under the influence of an external electric field, the mobile charge carriers are distorted near the multiple interfaces and junctions present in the composite material. These interfaces increase the Debye relaxation process, which also helps to improve the EM absorption characteristics. Increasing the content of Fe_3_O_4_/MXene will increase the electron annulus from Fe^+2^ and Fe^+3^, thereby improving the conductivity of the composite material. The best balance between dielectric constant and permeability can improve the impedance-matching characteristics of the absorber [49]. In addition, the hierarchical structure of TPFM composite materials can cause multiple reflections of electromagnetic waves. The *SE_A_*-to-*SE_R_* ratio of the 5 mg/mL MXene increased from 1.82 to 5.13 (Figure S4), indicating that the primary mechanism of the TPFM composites is absorption attenuation.

## 4. Conclusions

To prepare EMI shielding materials with ultrathin thickness, high durability, and excellent EMI *SE* characteristics, this work proposes a combination of improved electrospinning and coating to fabricate a conductive TPFM fiber film with superparamagnetism. The average thickness of the TPFM film is ~0.45 mm. The doping of PAN increases the bonding force between the conductive layer and the substrate. Moreover, this film is highly durable, with electromagnetic shielding performance maintained after repeated bending for 500 cycles. The electrospinning method allows a high loading of Fe_3_O_4_NPs to be easily and efficiently dispersed on the fibers, and the synergy of these magnetic nanoparticles with MXene provides an excellent electromagnetic shielding effect of up to 32.5 dB. The lightweight and stable mechanical properties of this film mean that it is suitable for use in flexible wearable devices and wireless human–computer interaction applications.

## Data Availability

Data presented in this article is available on request from the corresponding author.

## Supplementary Materials

The following are available online at https://www.mdpi.com/article/10.3390/nano12010020/s1, Figure S1. (a,b) SEM image of the multi-layer MXene and MXene after exfoliation, (c) The Tyndall effect of few layer MXene solution, Figure S2. (a–c) SEM image and element mapping images of TPFM, respectively, Figure S3. Stress-strain curves of the TPF-5 mg MXene with different Fe3O4NPs content, 0 wt%, 3 wt%, 6 wt% and 12 wt% respectively, Figure S4. The ratio of average SEA and SER of TPFM films as a function of Fe3O4NPs/MXene loading.
