# Supplementary material for: Interfacing MXene Flakes on a Magnetic Fiber Network as a Stretchable, Flexible, Electromagnetic Shielding Fabric
Source: Nanomaterials (Basel). 2021 Dec 22;12(1):20. doi: 10.3390/nano12010020 (PMC8746703; doi:10.3390/nano12010020)
Supplement: Supplementary file 1 [file nanomaterials-12-00020-s001.zip › nanomaterials-1484244-supplementary.pdf]

nanomaterials

Supplementary Material

Interfacing MXene Flakes on a Magnetic Fiber Network as a Stretchable, Flexible, Electromagnetic Shielding Fabric

Zhen Miao, Xiaohong Chen *, Honglei Zhou, Ping Liu, Shaoli Fu, Jiajie Yang, Yuhang Gao, Yupeng Ren, and Dong Rong

School of Materials Science and Engineering, University of Shanghai for Science and Technology, Shanghai 200000, China; 193742704@st.usst.edu.cn (Z.M.); 193742699@st.usst.edu.cn (H.Z.); 193742700@st.usst.edu.cn (P.L.); 171380130@st.usst.edu.cn (S.F.); 192432667@st.usst.edu.cn (J.Y.); 193742737@st.usst.edu.cn (Y.G.); 192432629@st.usst.edu.cn (Y.R.); 193742742@st.usst.edu.cn (D.R.)

* Correspondence: 193742705@st.usst.edu.cn

Citation: Miao, Z.; Chen, X.; Zhou, H.; Liu, P.; Fu, S.; Yang, J.; Gao, Y.; Ren, Y.; Rong, D. Interfacing MXene Flakes on Magnetic Fiber Network as a Stretchable Flexible Electromagnetic Shielding Fabric. *Nanomaterials* **2021**, *11*, x. https://doi.org/10.3390/nano12010020

Academic Editor(s): Ioan Bica

Received: 12 November 2021
Accepted: 16 December 2021
Published: 22 December 2021

Publisher's Note: MDPI stays neutral with regard to jurisdictional claims in published maps and institutional affiliations.

Copyright: © 2021 by the authors. Submitted for possible open access publication under the terms and conditions of the Creative Commons Attribution (CC BY) license (https://creativecommons.org/licenses/by/4.0/).

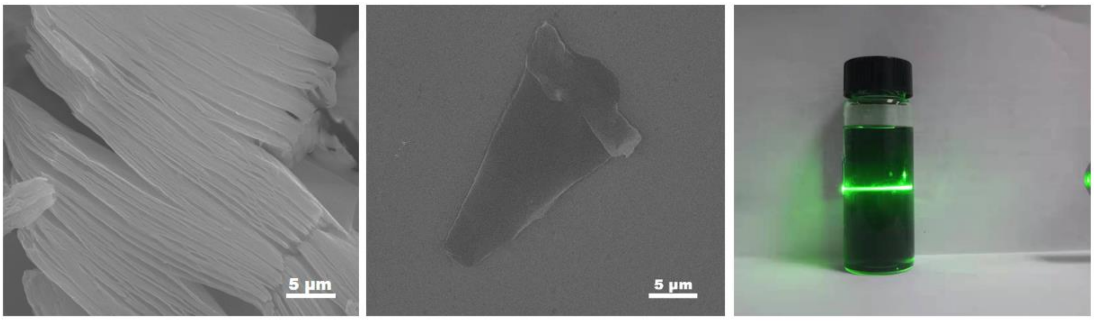

a b c

Figure S1. (**a**,**b**) SEM image of the Multi layer Mxene and MXene after exfoliation; (**c**) The Tyndall effect of few layer mxene solution.

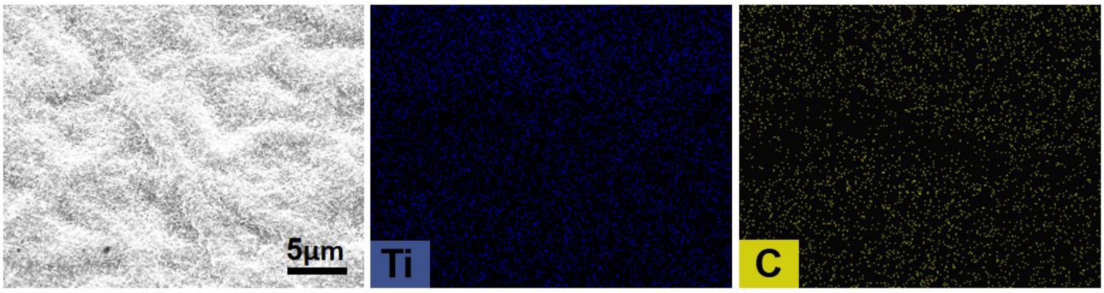

a b c

Figure S2. (a-c) SEM image and element mapping images of TPFM, respectively.

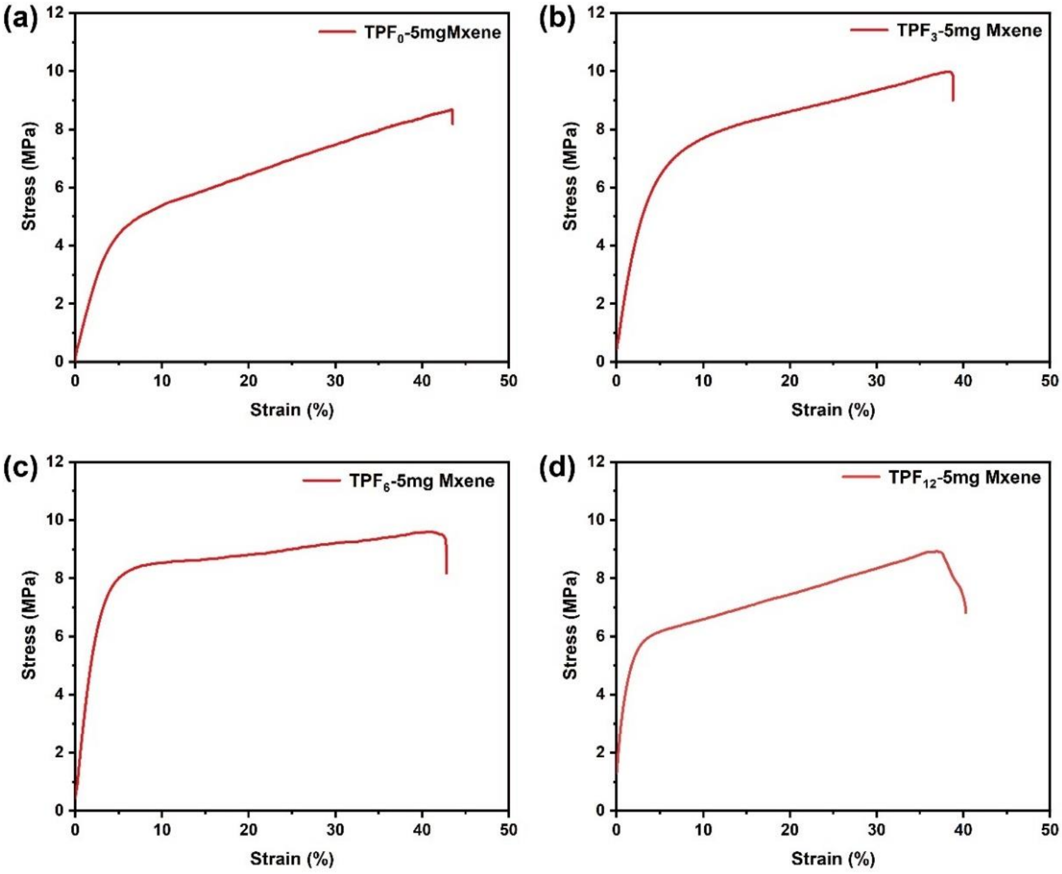

Figure S3. (a–d) Stress-strain curves of the TPF-5 mg MXene with different Fe_3O_4NPs content, 0 wt%, 3 wt%, 6 wt% and 12wt% respectively.

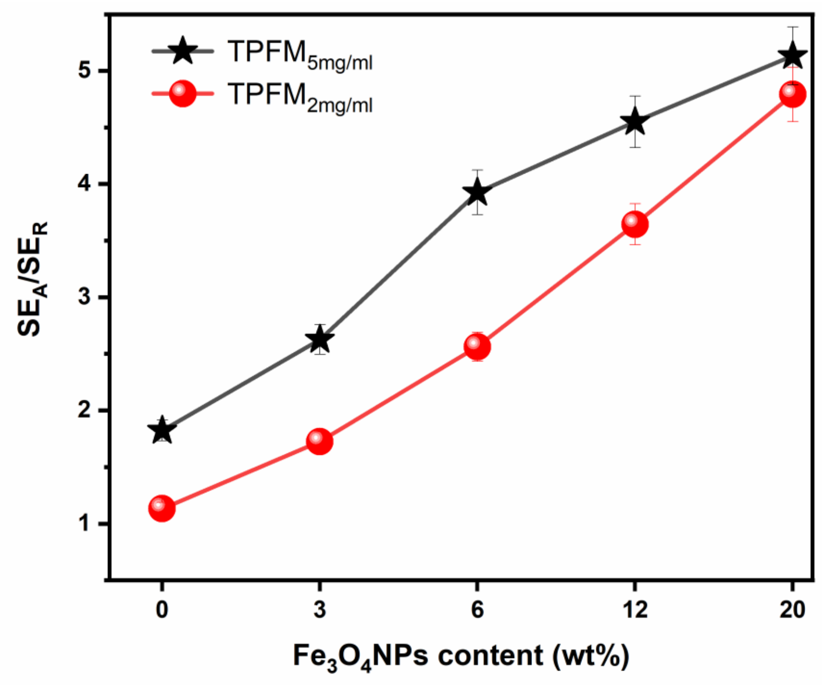

Figure S4. The ratio of average SE_A and SE_R of TPFM films as a function of Fe_3O_4NPs/MXene loading.
